# Effect of induced diabetes on morphometric indexes of the cerebellar cortex and gene expression in C57BL mice

**DOI:** 10.22038/IJBMS.2023.71172.15457

**Published:** 2023

**Authors:** Somayeh Sharifi, Masoud Golalipour, Soraya Ghafari, Razieh Safari, Mohammad Jafar Golalipour

**Affiliations:** 1 Department of Anatomical Sciences, Faculty of Medicine, Golestan University of Medical Sciences, Gorgan, Iran; 2 Cellular and Molecular Research Center, Department of Biology, Faculty of Technology, Golestan University of Medical Sciences Gorgan, Iran; 3 Department of Embryology and Histology, Golestan University of Medical Sciences, Gorgan, Iran; 4 Gorgan Congenital Malformations Research Center, Golestan University of Medical Sciences, and Gorgan, Iran; 5 Gorgan Congenital Malformations Research Center, Department of Anatomical Sciences, Golestan University of Medical Sciences, and Gorgan, Iran

**Keywords:** BDNF, Cerebellum, Diabetes, PAX7, Purkinje cell, SYNCAM1, SYP

## Abstract

**Objective(s):**

Diabetes is a metabolic disorder that affects the development of the central nervous system and plays an important role in learning and memory. Diabetes increases the reactive oxygen species (ROS) level in cells and changes the expression of several genes, including SYP, BDNF, PAX7, and SYNCAM1, through the FOXO transcription factor. This study was done to assess the effect of diabetes on morphometric indexes of the cerebellar cortex and gene expression in mice.

**Materials and Methods:**

Diabetes was induced in twelve adult, male C57BL mice using an injection of streptozotocin. After two months, the mice were dissected, and the cerebellum was stored for further analysis. For the morphometric analysis, tissue sections were stained with cresyl violet and examined with a light microscope. For gene expression analysis, the RNA was extracted, and cDNA was synthesized. The mRNA levels of SYP, BDNF, PAX7, and SYNCAM1 genes were measured by the real-time PCR method.

**Results:**

The thickness of the molecular layer and Purkinje layer, and the number of Purkinje and granular cells in the diabetic group were significantly reduced compared to controls *P*<0.0 1). The area, perimeter, and diameter of Purkinje cells in the diabetic group were significantly reduced compared to controls *P*<0.0 1). The expression of PAX7, SYP, and BDNF genes of the diabetic group was significantly reduced. However, SYNCAM1 expression in the cerebellum of the diabetic group was significantly increased compared to controls (*P*<0.05).

**Conclusion:**

Induced diabetes in mice can decrease the expression of memory-related genes in the cerebellum. Also, these genes affect the morphology and thickness of the cerebellum.

## Introduction

Diabetes is one of the most common complex chronic metabolic disorders, characterized by a deficiency of insulin hormone and insulin resistance. Reducing insulin secretion by the pancreas or insulin resistance in diabetes causes an enhancement in blood glucose levels. It changes the body’s metabolism of protein, lipids, and carbohydrates (1-4). Long-term complications of diabetes affect all systems and organs of the body, including the central nervous system. which leads to chronic neurological disorders by physiological and biochemical changes in nerve cells (5, 6). Therefore, diabetes leads to disorders, including learning and memory disorders (7-9). Hyperglycemia increases the production of reactive oxygen species (ROS) and reduces neurogenesis. Elevated levels of ROS lead to increased oxidative stress, inflammation, and death of neurons (10). The cerebellum is a part of the central nervous system located in the posterior cranial fossa and is involved in the evolution of language and thought (11, 12). Different cells in the cerebellum have essential roles in the execution of well-timed and coordinated movements, learning, and memory (13-16). ROS production due to diabetes could damage the cerebellum cells. In addition, ROS prevents memory-related gene expression by inhibiting FOXO family transcription factors. We hypothesized that diabetes increases the ROS level which affects the cerebellum structure and changes the expression of memory-related genes in the cerebellum. In the present study, we tried to investigate the effects of diabetes on the structural and functional damage of the cerebellum and memory-related genes, including SYP, BDNF, and PAX7 genes in the cerebellum.

## Materials and Methods

***Animals***

This experimental study was conducted on 12 adult, male C57BL mice cerebellum samples (frozen samples of the previous study). The mice cerebellum samples were randomly divided into two diabetic (n=6) and control (n=6) groups. Mice were housed in individual cages under temperature-controlled standard conditions, which included the usual circadian rhythm (12 hr of light and 12 hr of night), an average temperature of 25 °C, and access to adequate food pellets and drinking water. Mice were randomly divided into two diabetic (n=6) and control (n=6) groups. All animal experiments were approved by the institutional animal care and use committee of the Golestan University of Medical Sciences, Gorgon, Iran. (Ethical Code: IR.UOZ.REC.1401.009).

***Induction of diabetes***

According to a previous study, the blood glucose level of all adult male mice was determined by an Accu-Chek Active system (Roche, Mannheim, Germany) (17). Then adult male mice (n=6, 9 weeks) with normal blood glucose levels were randomly injected with streptozotocin (STZ) under the name of the diabetic group. STZ was injected as an intraperitoneal solution in sodium citrate during two stages with a time interval of 5 days at a dose of 150 mg/kg of body weight (18). Blood glucose was measured 72 hr after injection. Mice with blood glucose levels above 250 mg/dl were considered diabetic (19). Mice in the control group were injected with citrate buffer.

***Dissection of the cerebellum***

Two months after the induction of diabetes, the cerebellum of the mice was surgically removed from the skull area. The samples of the cerebellum of mice of both the diabetic and the control groups were taken out from the skull and transferred to a -80 °C freezer. Half of the cerebellar samples from both the diabetic and control groups were used to extract the RNA, to measure the changes in the expression of genes, and the other half to be used to assess the morphometric changes of the cerebellum.

***Morphometric analysis***

Samples of the cerebellum were fixed in 10% neutral-buffered formalin for 48 hr (20). All samples were dehydrated with ascending alcohol grades, cleared in xylene, and embedded in paraffin. Horizontal sections were cut to a thickness of 6 μm and stained with cresyl violet for histological examination (21). Each cerebellum section saved 10–15 images for further analysis. The number of Purkinje and granular cells was counted in an area equal to 30,000 square μm of the Purkinje and granular layers. The thickness of the molecular, Purkinje, and granular layers and white matter in the cerebellar lobes were recorded. In addition, the area, perimeter, large diameter, and small diameter of cells were measured. The data relating to each section were collected and recorded in anterior-posterior order and separately for the studied groups.

***Quantitative real-time PCR (RT-qPCR)***

For cDNA synthesis, the first-strand cDNA synthesis kit (Sinnaclon, Tehran, Iran) was used. For each sample, 1 μg of each RNA sample was used in the reaction. In real-time PCR reaction, 1 μl of cDNA was used in an SYBR green qPCR master mix 2X (Yekta-Tajhiz-Azma, Tehran, Iran). The cycling program was as follows: first, the denaturation step at 95 °C for 5 min. second, 40 cycles of annealing for 10 sec at 95 °C, and extension at 62 °C (SYP, BDNF, SYNCAM1), 60 °C (PAX7), for 30 sec. The final step was extension at 72 °C for 40 sec. Melting curve analysis was done in temperature ranges from 95 °C to 60 °C for 20 sec. The B-actin was used as the internal control. The sequence of primers is presented in Table 1.

***Statistical analysis***

All data were analyzed using Graph Pad Prism software (version 9). The normality of data was tested by a one-sample Kolomogrov-Smirnov test. The variance analysis method was used to test the equality of means. The multiple unpaired T-tests method was used to calculate the average thickness of the cerebellar layer and the size of the large and minor diameters of Purkinje cells. The T-test was used to calculate the average number of Purkinje cells and the number of granular cells. The one-sample T-test method was used to calculate the average area and perimeter of Purkinje cells. To calculate the mRNA expression of SYP, BDNF, PAX7, and SYNCAM1, the relative expression method (2-(∆∆CT)) was used in the calibrator-normalized method. SYP, BDNF, PAX7, and SYNCAM1 expressions were presented as mean±SEM. To observe statistical differences between different groups, T-tests were performed. *P*-value<0.0**5** was considered statistically significant.

## Results

***Morphological changes in cerebellar layers and cells***

**Figure 1:**
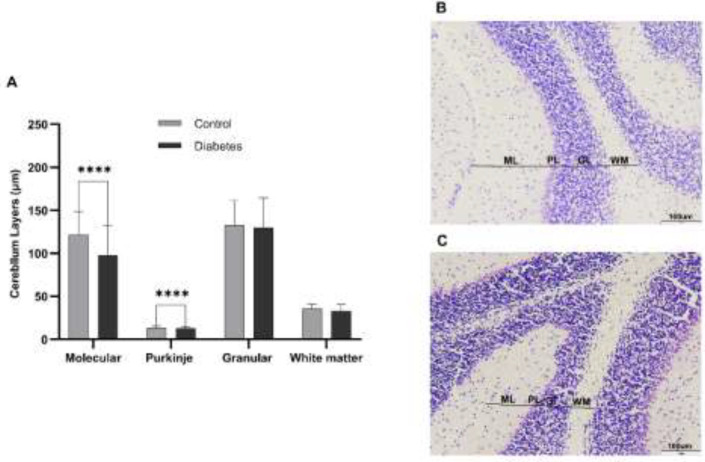
Thickness of molecular, Purkinje, and granular layers and white matter in the cerebellum of adult, male mice of diabetic and control groups

The thickness of the molecular layer of the cerebellum was significantly reduced in the diabetic group (average of 97.56 μm) in comparison with the control group (average of 121.52 μm) *P*<0.0000) (Figure 1). Also, the thickness of the Purkinje cell layer of the cerebellum was significantly reduced in the diabetic group (average of 19.64 μm) compared to controls (average of 23.17 μm) *P*<0.0000) (Figure 1). Although the thickness of the granular layer and white matter of the diabetic group was decreased, it was not significant (Figure 1). 

**Figure 2:**
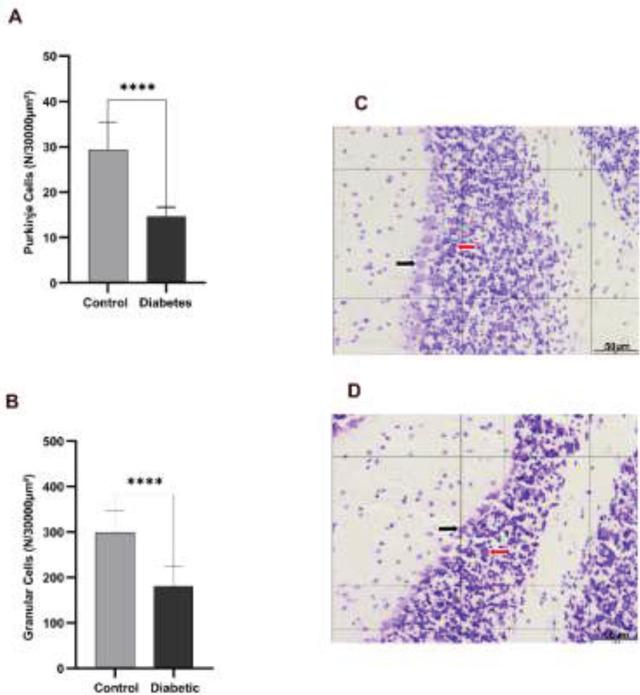
Number of Purkinje and granular cells in the cerebellum of adult, male mice of diabetic and control groups

The number of Purkinje cells (area = 30000 μm²) was significantly lower in the diabetic group than in the control group (14.69 μm² vs 29.35 μm²) *P*<0.0001) (Figure 2). Also, the number of granular cells (area = 30000 μm²) was significantly reduced in the diabetic group (average of 180.96 μm²) in comparison to controls (average of 299.46 μm²) *P*<0.0001) (Figure 2).

**Figure 3:**
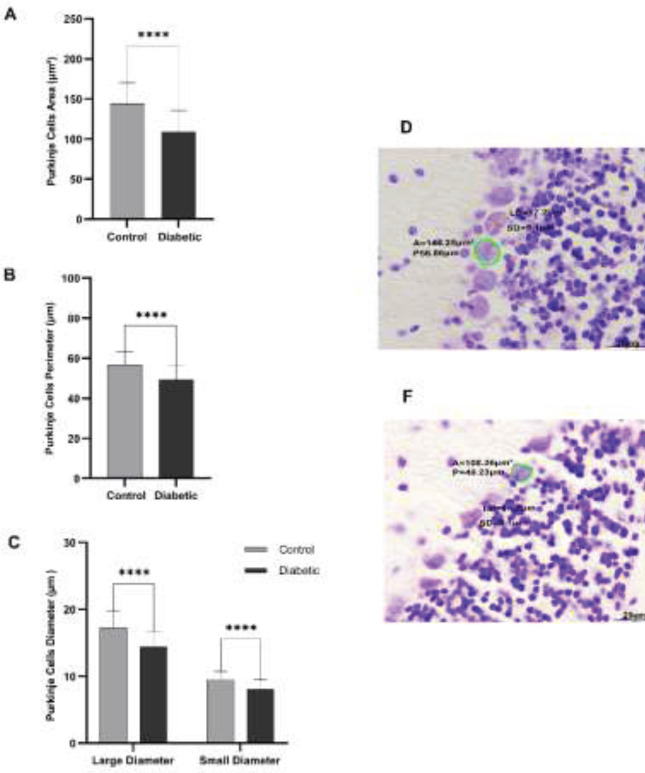
Area, perimeter, and large, small diameter circumference of Purkinje cells in the cerebellum of adult, male mice of the diabetic and control groups

The area of cerebellar Purkinje cells was significantly reduced in the diabetic group (average of 108.60 μm²) in comparison with controls (average of 144.15 μm²**) ***P*<0.0001) (Figure 3). The perimeter of cerebellar Purkinje cells was significantly reduced in the diabetic group compared to the controls (average of 49.23 μm vs 56.86 μm) *P*<0.0001) (Figure 3). Also, the thickness of the large diameter of the cerebellar Purkinje cells significantly reduced in the diabetic group (14.40 μm) in comparison with controls (17.26 μm) *P*<0.0000) (Figure 3). The thickness of the small diameter of cerebellar Purkinje cells was significantly lower in the diabetic group than in the control group (8.06 μm vs 9.45 μm) *P*<0.0001) (Figure 3). 

***Expression of memory-related genes in the cerebellum***

**Figure 4:**
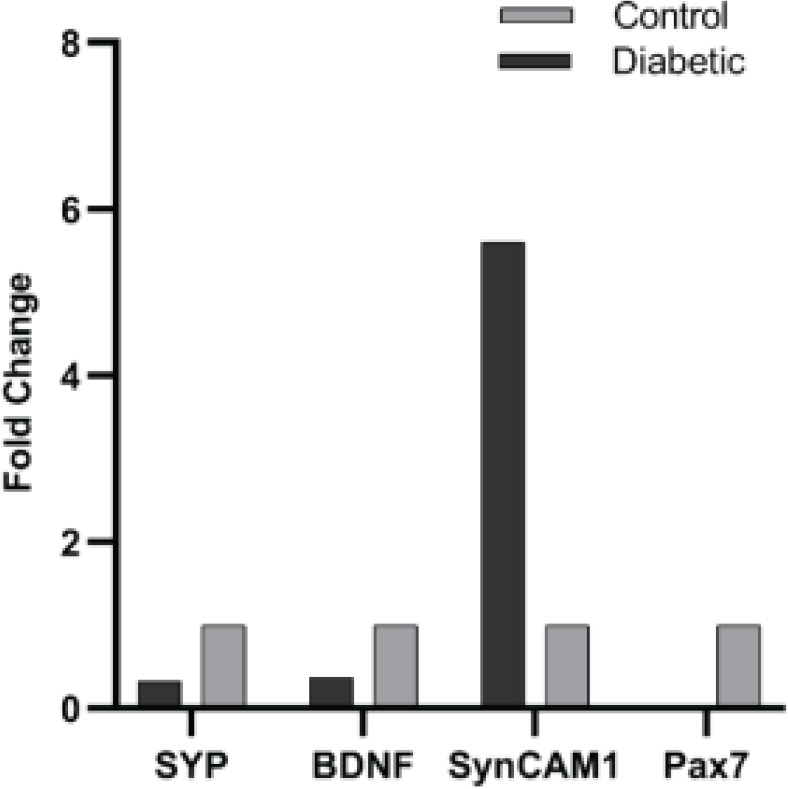
Expression levels of SYP, BDNF, PAX7, SYNCAM1 genes in the cerebellum of adult male mice of diabetic and control groups

In the present study, we used the real-time PCR method to investigate the morphometric correlation of the cerebellar cortex and changes in the expression of SYP, BDNF, PAX7, and SYNCAM1 genes in the cerebellum of diabetic adult male mice in the two diabetic and control groups. The SYP, BDNF, PAX7, and SYNCAM1 expression levels were normalized by internal control B-actin gene expression. Figure 4 shows the relative mRNA expression levels of SYP, BDNF, PAX7, and SYNCAM1 genes in the cerebellum of adult diabetic male mice. A significant reduction was observed in the induced diabetic group compared to the control group (*P*<0.05). SYP, BDNF, and PAX7 gene expression was reduced to 0.32, 0.36, and 0.001 fold in the diabetic group. However, the level of SYNCAM1 gene expression in the cerebellum of the diabetic group was significantly higher than in the controls (5.60) (*P*<0.05) (Figure 4).

**Table 1:** Sequence of primers used in real-time quantitative PCR for gene expression

***Gene ***	***Primers sequences (5'-3') ***
**SYP**	*TTGGCTTCGTGAAGGTGCTGCA (F)* *ACTCTCCGTCTTGTTGGCACAC (R) *
**BDNF**	*TGCAGGGGCATAGACAAAAGG (F) * *CTTATGAATCGCCAGCCAATTCTC (R) *
**SYNCAM1 **	*ACTTCTGCCAGCTCTACACGGA (F)* *CCCTTCAACTGCCGTGTCTTTC (R) *
**PAX7 **	*CTCAGTGAGTTCGATTAGCCG (F) * *AGACGGTTCCCTTTGTCGC (R) *
**B-actin **	*CATCCGTAAAGACCTCTATGCCAAC (F) * *ATGGAGCCACCGATCCACA (R) *

(A) The thickness of the cerebellar cortex layers and white matter in the diabetic and control groups. Values represent the means±SEM (n=12). The changes in the thickness of cerebellar layers, in control (B) and diabetic (C) mice, molecular layer of the outer layer of the cerebellum (ML), Purkinje layer of the middle layer of the cerebellum (PL), granular layer of the inner layer of the cerebellum (GL), the white matter of the cerebellum (WM) (staining: cresyl violet, magnification 200x).

The number of Purkinje cells (black arrow) and cerebellar granular cells (red arrow) in the diabetic and control groups. (A) thickness of the changes in the number of cerebellar Purkinje cells, (B) thickness of the changes in the number of cerebellar granular cells, control group (C), diabetic group (D). Values represent the means±SEM (n = 12) (staining: cresyl violet, magnification 400x).

The area (A), perimeter (B), and the large and small diameter (C) of the cerebellar Purkinje cells were significantly reduced in the diabetic group *P*<0.0001). Control group (D), diabetic group (F), the large diameter of Purkinje cells (yellow line: LD), small diameter (red line: SD) in figure (D) control group, and figure (F) diabetic group show a significant decrease. The area and perimeter (green circle) of Purkinje cells in Figure (D) control group and Figure (F) diabetic group show a significant decrease. Values represent the means±SEM (n=12) (staining: cresyl violet, magnification 1000x).

The expression levels of SYP, BDNF, and PAX7 genes in the cerebellum of the diabetic group were significantly decreased (*P*<0.05). The level of SYNCAM1 gene expression in the cerebellum of the diabetic group was significantly increased compared to the control group (*P*<0.05). Values represent the means±SEM (n=12).

**Figure 5:**
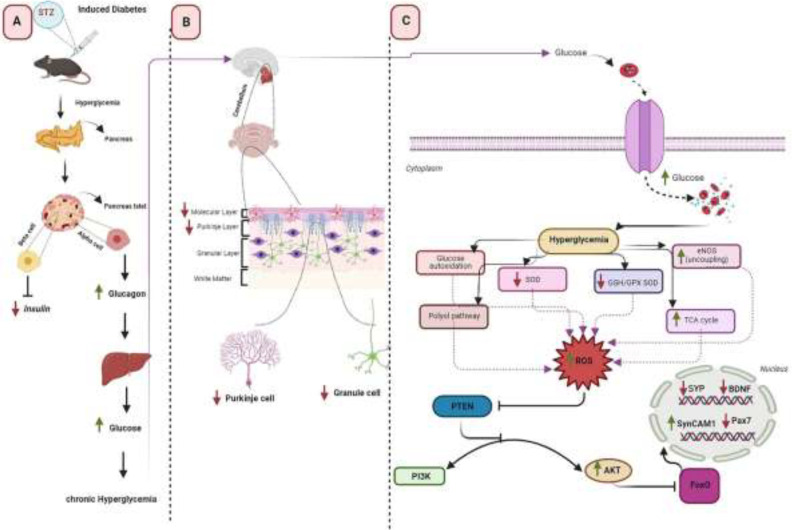
The possible pathway of the effect of diabetes on the expression of genes affecting memory in the cerebellum of the adult mice

## Discussion

Diabetes is one of the metabolic disorders, and the long-term complications of diabetes affect all systems and organs of the body, including the central nervous system. In the present study, we investigated the effects of diabetes on the structural damage of the cerebellum and memory-related (SYP, BDNF, PAX7, and SYNCAM1) genes. Our results showed that diabetes causes a significant change in the morphology of the Purkinje and granular cells of the cerebellum, as well as the thickness of the cerebellar layers. In addition, we have found that the expression of genes related to learning and memory decreased after the induction of diabetes.

The thickness of the molecular and Purkinje layers was decreased by 20% and 16%, respectively. However, no significant decrease was observed in the granular layers and white matter. Faizal *et al*.’s study on the effect of diabetes on the cerebellum of albino rats showed that long-term hyperglycemia leads to a decrease in the thickness of the cerebellar cortex layers (22). Also, Khaksary* et al*., in 2021, have reported that the thickness of the molecular, Purkinje, granular, and white matter layers decreases after diabetes (23). Our results also showed that the number, area, perimeter, and diameter of Purkinje and granular cells decreased. The reduction in Purkinje and granular cells was also reported in other studies (22-24). It seems that the decrease in the thickness of the cerebellar layers is caused by the decrease in the number of cells in those areas. The mechanism of diabetes effects on the cerebellar morphology may be due to the increased ROS production. A continuous increase in blood glucose levels leads to production of ROS, which leads to oxidative stress (25). Furthermore, ROS prevents memory-related gene expression by inactivating FOXO family transcription factors. Therefore, we assessed the effects of diabetes on the expression of FOXO-regulated memory-related genes, including SYP, BDNF, and PAX7 genes in the cerebellum. Our results showed that significant changes occurred in these genes’ expression levels. The role of SYP, BDNF, and PAX7 in memory and the effect of diabetes on the reduction of these genes have been reported in several studies (26-30). We proposed a possible mechanism for the effect of diabetes on memory loss in the cerebellum through ROS production and inhibition of FOXO-regulated genes (Figure 5).

## Conclusion

We have found a significant change in the cerebellum’s morphology and the expression of memory-related genes in diabetic mice. Our findings may be helpful to better understand the effects of diabetes on memory.

## Authors’ Contributions

All Authors contributed to concept and design of the study and approved the final version to be published. S S, S G, and R S were responsible for acquisition of data. MJ G and M G drafted the article or revised it critically for important intellectual content. MJ G assumed responsibility for the study. 

## Ethical Approval

The approval of the ethics committee of Golestan University, Iran (ethical code: IR.GOUMS.AEC.1401.010) was issued.

## Conflicts of Interest

We have no conflicts of interest.
